# Brazilian version of the Kerlan-Jobe orthopedic clinic shoulder and elbow score: translation and cross-cultural adaptation

**DOI:** 10.1590/1516-3180.2023.0139.03072024

**Published:** 2024-12-20

**Authors:** Felipe Marques da Silva, Raquel Lins de Sousa Lima, Gabriel Alves dos Santos, Germanna Medeiros Barbosa, Valéria Mayaly Alves de Oliveira, Danilo Harudy Kamonseki

**Affiliations:** I Undergraduate Physical Therapy student, Universidade Federal da Paraíba (UFPB), João Pessoa (PB), Brazil.; II Undergraduate Physical Therapy student, Universidade Federal da Paraíba (UFPB), João Pessoa (PB), Brazil.; III Undergraduate Physical Therapy student, Universidade Federal da Paraíba (UFPB), João Pessoa (PB), Brazil.; IV Professor, Postgraduate Program in Rehabilitation Sciences (PPGCREAB), Health Sciences College of Trairi, Universidade Federal do Rio Grande do Norte (UFRN), Santa Cruz (RN), Brazil.; V Professor, Department of Physical Therapy, Universidade Federal da Paraíba (UFPB), João Pessoa (PB), Brazil.; VI Professor, Department of Physical Therapy, Universidade Federal da Paraíba (UFPB), João Pessoa (PB), Brazil.

**Keywords:** Athlete, Patient Reported Outcome Measure, Shoulder pains, Elbow joints, Translatings, Sport Medicine, Questionnaire, Arms, Physical Therapy

## Abstract

**BACKGROUND:**

The Kerlan-Jobe Orthopedic Clinic (KJOC) Shoulder and Elbow Score is commonly used to assess the functional status of athletes with conditions affecting the shoulder and elbow. However, a Brazilian Portuguese version of the KJOC questionnaire is currently unavailable.

**OBJECTIVES:**

This study aimed to develop a Brazilian Portuguese version of the KJOC questionnaire.

**DESIGN AND SETTING:**

This translation and cultural adaptation study was conducted at the Federal University of Paraíba, Brazil.

**METHODS:**

The procedures adopted in this study followed guidelines recommending translation by two independent translators, synthesis of the translations, back-translation performed by two native English-speaking translators, analysis by an expert committee, and pre-testing. The Portuguese version was tested with 32 athletes to assess their understanding of the assessment tool. Items were deemed adequate if they were understood by at least 90% of the athletes.

**RESULTS:**

The terms and expressions of some original items were modified to achieve better comprehensibility in the Brazilian context. No further modifications were necessary after the pre-test; all terms were comprehensible to over 90% of athletes.

**CONCLUSION:**

The translation and cultural adaptation of the KJOC questionnaire into Portuguese were completed, resulting in a Brazilian version of the scale. Further studies are needed to evaluate the reliability and validity of this scale.

## INTRODUCTION

Musculoskeletal injuries are common among athletes, resulting from high physical demands, including movements with high load and speed, abrupt changes in direction, and physical contact with other athletes.^1,2^ In overhead athletes (athletes participating in a sport that requires movements of the upper arm over the head), the decelerating phase of throwing gestures, which generates shoulder distraction forces up to 1.5 times the athlete’s body weight, and the repetitive tensile loading in posterior structures may cause structural changes in the shoulder.^3^ In addition, the rotational speed of the arm may exceed 7000°/s during a throw with a distraction force of 947 N (108% body weight) at the glenohumeral joint.^4^ Other factors may be associated with injury occurrence, such as age, sex, history of previous injuries, poor physical fitness, insufficient muscle strength, improper training, increased training volumes, short rest periods, type of training surface, and lack of preventive measures.^5,6^

Upper limb injuries have a high incidence and prevalence in overhead athletes, accounting for approximately 8 to 13% of all sports-related injuries and approximately 75% of injuries associated with sports involving throwing. The shoulder and elbow joints are most commonly affected.^7,8,9,10^ Shoulder pain affects approximately 50% of baseball players during their careers,^11^ and 44% of volleyball players.^7^ The prevalence of shoulder injuries among handball players is high, ranging from 19.6%, in Norway,^12^ to 36% in Iran.^13^ Furthermore, the reported incidence of shoulder injuries is 44% in Brazil.

These conditions can directly affect athletes’ performance, causing clinically significant dysfunction that may result in reduced dexterity, missed training sessions and games, loss of sponsorships, and even early retirement.^7,9^ The evaluation of shoulder and elbow injuries and its impact on function and sports performance is crucial. Proper assessment can assist therapists in characterizing athletes, identifying functional deficits, analyzing prognosis, establishing criteria for return to sport, and verifying treatment effectiveness.^8,14^

The main patient-reported outcome measures for evaluating shoulder function in athletes are the Kerlan-Jobe Orthopedic Clinic (KJOC) Shoulder and Elbow Score,^15,16,17,18^ Disabilities of the Arm, Shoulder and Hand (DASH) questionnaire,^19^ Functional Arm Scale for Throwers (FAST),^8^ Penn Shoulder Score,^20^ and the American Shoulder and Elbow Surgeons Evaluation Form (ASES).^21^ However, the DASH questionnaire and ASES have few questions that assess the specificities of overhead sports, and the FAST focuses only on throwing athletes and has not yet been translated into Brazilian Portuguese.^8,22,23^

The KJOC questionnaire was developed in English and is considered an important tool for assessing the shoulder and elbow in overhead athletes, being specifically validated in this population.^14,22^ The KJOC questionnaire is a patient-reported outcome measure that assesses athletes’ performance, symptoms, and function, including sports-related interpersonal interactions. The final score enables sensitive observation of changes in the function and performance of overhead athletes.^14,17,24^

Although it has been translated and validated for use in various countries such as Germany,^25^ Spain,^15^ Finland,^14^ Norway,^17^ Greece,^26^ Korea,^18^ Turkey,^27^ and Italy,^16^ and has already been used as a tool in randomized studies,^28^ quasi-experimental studies,^24^ and longitudinal studies,^29^ this questionnaire has not been translated and culturally adapted for use in Brazil.^30^

## OBJECTIVES

The current study aimed to translate and culturally adapt the KJOC questionnaire into Brazilian Portuguese.

## METHODS

### Study Design

This cross-sectional study followed guidelines for translation and cultural adaptation.^31,32,33^ The study was approved by the Research Ethics Committee (CAAE No. 67423323.3.0000.5188, on April 25, 2023) of the Universidade Federal da Paraíba (UFPB). All individuals who agreed to participate signed an informed consent form.

### Participants

This study included athletes of both sexes, aged between 18 and 60 years, with at least one year of competitive practice in an overhead sport, who were either asymptomatic or had shoulder or elbow pain. Asymptomatic athletes were included in this study based on previous reports demonstrating that the KJOC questionnaire can assess functional status in asymptomatic athletes.^29,37^ Participants were required to maintain a regular regimen with training at least twice a week and participate in sports for competitive purposes, which involved participation in championships and tournaments, regardless of the level (college, regional, state, or national). ^34,35,36^ Athletes with cognitive impairments that would preclude them from completing the assessment were excluded from the study.

### Procedures

The translation and adaptation process of the KJOC questionnaire for Brazilian athletes was based on the following steps: 1) translation, 2) synthesis of translations, 3) back-translation to the original language, 4) committee analysis, and 5) pre-testing.^31,32,33^

The author of the original version of the KJOC questionnaire authorized this study via e-mail. The KJOC questionnaire was translated by two independent translators who were fluent in both Brazilian and English. The first translator, with expertise in the health sciences, was informed of the research objectives. The second translator, without expertise in health sciences, was not informed of the concepts being assessed. Each translator produced versions of the questionnaire, V1 and V2. These versions were read and discussed by both the translators and the researchers to synthesize V1 and V2 into a single version (V3).

V3 was translated back into English by two independent native English-speaking translators fluent in Portuguese, resulting in two independent versions: B1 and B2. The back translators were blinded to the original version of the questionnaire and did not have a medical background. Subsequently, a committee composed of back-translators, translators, two physical therapy students, one physiotherapist, and two professors with PhDs in physical therapy discussed the differences between all versions (V1, V2, V3, B1, and B2) and the original questionnaire, aiming to produce the final version used in the pre-testing phase. The discussions identified sentences that required modifications and were rewritten to enhance the comprehensibility and semantic, conceptual, idiomatic, and cultural equivalence of the tool, resulting in the version used in the pre-test (pre-test version).

The pre-test was conducted with 32 athletes from different overhead sports. The sample size was determined based on the recommendation of Beaton et al. ^32^, which considers 30 individuals to be adequate. The athletes completed the KJOC questionnaire without further explanation beyond the initial instructions to prevent any potential influence on their responses. Athletes were instructed to: 1) read and respond to the pre-test version; 2) indicate if they understood the questions (Yes or No question); 3) and provide suggestions for each item, if any. The items that obtained a “non-understanding” level above 10% were reformulated to create the final version, which was then sent to the author of the KJOC questionnaire for approval.^38,39,40^

### Scoring Calculation

The KJOC questionnaire consists of 19 items divided into two sections. The first section, comprising 9 items, included questions about the history of the current injury, a description of the current competition level, and the current state of the arm. The second section includes 10 questions, measured with a ten-centimeter Visual Analog Scale (VAS), about physical functioning, playing, and practice conditions, and specific questions regarding the competition level in the relevant sport. The total score was obtained by summing the scores obtained from all items in the second section, resulting in a total score between 0 and 100 points. Higher scores indicate better arm function.^14,18^

## RESULTS

This study included 32 overhead athletes, and their characteristics are shown in **Table 1**. In the translation stage, the committee analyzed and discussed all questions from versions V1 and V2 to formulate the consensus version, V3 (**Table 2**).

**Table 1:** Characteristics of the included individuals

Characteristics	n = 32
**Sex, n (%)**	
Male	22 (69)
Female	10 (31)
**Age, years**	31.8 ± 10.53
**Body Mass Index, kg/m^2^**	77.5 ± 20.35
**Years of Sports Practice**	13.3 ± 11.22
**Sport Modality, n (%)**	
Baseball	17 (53)
Volleyball	14 (44)
Handball	1 (3)
Asymptomatic individuals	11 (34.4%)
Symptomatic individuals	21 (65.6 %)
**Educational Level, n (%)**	
Elementary School	1 (3)
High School	12 (38)
Higher Education	17 (53)
Postgraduate	2 (6)

Continuous data were described as mean ± standard deviation, and categorical data were described as frequency (%).

**Table 2:** Comparison of translations and development of the consensus version

Original	V1 e V2	V3
Name... Age... Sex... Dominant Hand (R) (L) (Ambidextrous)... Date of Examination... Sport... Position... Years Played	V1 - Nome... idade... sexo... mão dominante (D) (E)... (Ambidestro)... Data do teste... Esporte... Posição... Há quanto tempo pratica... V2 - Nome... Idade... Sexo... Mão dominante... (D) (E) (Ambidestro)... Data do exame... Esporte... Posição... Anos em ativa	Nome... idade... sexo... mão dominante (D) (E) (Ambidestro)... Data do teste... Esporte... Posição… Anos em ativa
Please answer the following questions related to your history of injuries to YOUR ARM ONLY:	V1 - Por favor responda às seguintes questões relacionadas ao seu histórico de lesões, BRAÇO APENAS. V2 - Por favor responda às seguintes questões relacionadas ao seu histórico de lesões APENAS DO SEU BRAÇO.	Por favor responda às seguintes questões relacionadas ao seu histórico de lesões APENAS DO SEU BRAÇO.
1. Is your arm currently injured?	V1 - Seu braço está lesionado atualmente? V2 - Seu braço está lesionado no momento?	1. Seu braço está lesionado atualmente?
2. Are you currently active in your sport?	V1 - Você está ativo(a) no seu esporte atualmente? V2 - Você está ativo no seu esporte?	2. Você está ativo(a) no seu esporte atualmente?
3. Have you missed game or practice time in the last year due to an injury to your shoulder or elbow?	V1 - Você já perdeu alguma partida ou treinamento, no último ano, por causa de uma lesão no ombro ou cotovelo? V2 - Você perdeu algum jogo ou treino no último ano devido a uma lesão no seu ombro ou cotovelo?	3. Você perdeu alguma partida (jogo) ou treino, no último ano, devido a uma lesão no seu ombro ou cotovelo?
4. Have you been diagnosed with an injury to your shoulder or elbow other than a strain or sprain? If yes, what was the diagnosis?	V1 - Você já foi diagnosticado com alguma lesão no ombro ou cotovelo além de tensão muscular ou torção? Se sim, qual foi o diagnóstico? V2 - Você foi diagnosticado com uma lesão no seu ombro ou cotovelo além de um estiramento ou entorse? Se sim, qual foi o diagnóstico?	4. Você já foi diagnosticado com alguma lesão no ombro ou cotovelo além de estiramento ou entorse? Se sim, qual foi o diagnóstico?
5. Have you received treatment for an injury to your shoulder or elbow? If yes, what was the treatment? (Check all that apply): Rest... Therapy... Surgery (please describe)	V1 - Você já recebeu algum tratamento para lesões em seu ombro ou cotovelo? Se sim, qual foi o tratamento? (Marque todos que se aplicam)... Descanso... Terapia... Cirurgia (por favor descreva) V2 - Você recebeu tratamento para alguma lesão no seu ombro ou cotovelo? Se sim, qual foi o tratamento? (Marque todas que se aplicam)... a. Descanso... b. Terapia... c. Cirurgia (por favor especifique)	5. Você já recebeu algum tratamento para lesões em seu ombro ou cotovelo? Se sim, qual foi o tratamento? (Marque todos que se aplicam)... a. Descanso... b. Terapia... c. Cirurgia (por favor especifique)
Please describe your level of competition in your current sport: (Use Professional Major League, Professional Minor League, Intercollegiate, High School as the choices)	V1 - Por favor descreva sua categoria de competição no seu esporte atual: (Use Profissional das Grandes Ligas, Profissional da Segunda-divisão, Intercolegial, Ensino Médio como as opções) V2 - Por favor, descreva seu nível de competição no seu esporte atual: (Use: Liga Principal Profissional, Liga Menor Profissional, Intercolegiado, Escolar)	Por favor, descreva seu nível de competição no seu esporte atual: (Use como opções: Profissional das Grandes (principais) Ligas, Profissional da Segunda-divisão, Intercolegial, Escolar).
6. What is the highest level of competition you’ve participated at?	V1 - Qual a categoria de competição mais alta que você já participou? V2 - Qual é o maior nível em que você já competiu?	6. Qual é o maior nível de competição em que você já participou?
7. What is your current level of competition?	V1 - Qual sua categoria atual? V2 - Qual o nível em que você está competindo atualmente?	7. Qual o nível em que você está competindo atualmente?
8. If your current level of competition is not the same as your highest, do you feel it is due to an injury to your arm?	V1 - Se a sua categoria atual não é a mesma que a categoria mais alta que já atingiu, você sente que seja por causa de uma lesão no braço? V2 - Se o seu nível de competição não é o mesmo que o maior em que você já competiu, você sente que é devido à lesão no seu braço?	8. Se o seu nível atual de competição não é o mesmo que o maior em que você já competiu, você sente que é devido à lesão no seu braço?
Please check the ONE category only that best describes your current status: Playing without any arm trouble... Playing, but with arm trouble... Not playing due to arm trouble	V1 - Por favor marque apenas UMA opção que melhor descreva sua situação atual: Jogando sem nenhum problema no braço... Sem jogar por causa de um problema no braço... Jogando, mas com problema no braço. V2 -Por favor marque a ÚNICA categoria que descreve melhor seu estado atual: Jogando sem nenhum problema no braço... Jogando, mas com problema no braço... Sem jogar devido a um problema do braço.	Por favor marque apenas UMA opção que melhor descreva sua situação atual: Jogando sem nenhum problema no braço... Jogando, mas com problema no braço... Sem jogar por causa de um problema do braço
Section II		
Instructions to athletes: The following questions concern your physical functioning during game and practice conditions. Unless otherwise specified, all questions relate to your shoulder or elbow. Please answer with an X along the horizontal line that corresponds to your current level.	V1 - Instrução para os atletas: As perguntas a seguir são acerca de seu funcionamento físico durante jogos e práticas. A menos que caso contrário seja especificado, todas as perguntas são relacionadas aos seus ombros e cotovelos. Por favor, responda com um X sobre a linha horizontal da forma que você ache que melhor corresponde ao seu nível atual. V2 - Instruções para atletas: As questões a seguir se referem ao seu funcionamento físico durante jogos e treinos. Todas as questões estão relacionadas ao seu ombro ou cotovelo, a não ser que especifiquem o contrário. Por favor responda com um X no espaço da linha que corresponde ao seu nível atual.	Instruções para atletas: As questões a seguir se referem ao seu funcionamento físico durante jogos e treinos. Todas as questões estão relacionadas ao seu ombro ou cotovelo, a não ser que seja especificado o contrário. Por favor, responda com um X sobre o espaço da linha horizontal que melhor corresponde ao seu nível atual.
1. How difficult is it for you to get loose or warm prior to competition or practice? Never feel loose during games or practice; Normal warm-up time	V1 - O quão difícil é para você relaxar ou se aquecer antes de uma competição ou prática? Nunca me sinto relaxado(a) durante jogos ou práticas; Tempo normal de aquecimento. V2 - Quão difícil é para você aquecer ou se soltar antes de uma competição ou treino? Nunca se sente solto durante jogos ou treino; Tempo normal de aquecimento.	1. Quão difícil é para você aquecer ou se soltar (relaxar) antes de uma competição ou treino? Nunca se sente solto (relaxado(a)) durante jogos ou treino; Tempo normal de aquecimento
2. How much pain do you experience in your shoulder or elbow? Pain at rest; No pain with competition	V1 - Quanta dor você sente em seu ombro ou cotovelo? Dor no descanso; Sem dor na competição. V2 - Quanta dor você sente no seu ombro ou cotovelo? Dor mesmo descansando; Sem dor em competições	2. Quanta dor você sente em seu ombro ou cotovelo? Dor no descanso; Sem dor na competição
3. How much weakness and/or fatigue (ie, loss of strength) do you experience in your shoulder or elbow? Weakness or fatigue preventing any competition; No weakness, normal competition fatigue	V1 - Quanta fraqueza e/ou fadiga (ou seja, perda de força) você experiencia em seu ombro ou cotovelo? Fraqueza ou fadiga impedindo qualquer competição; Sem fraqueza, fadiga de competição normal V2 - Quanta fraqueza e/ou fadiga (leia-se, perda de força) você sente no seu ombro ou cotovelo? Fraqueza ou fadiga que me impede de competir; Sem fraqueza, sinto uma fadiga normal de competição	3. Quanta fraqueza e/ou fadiga (ou seja, perda de força) você sente no seu ombro ou cotovelo? Fraqueza ou fadiga que me impede de competir; Sem fraqueza, sinto uma fadiga normal de competição.
4. How unstable does your shoulder or elbow feel during competition? “Popping out” routinely; No instability	V1 - Quão instável seu ombro ou cotovelo fica durante as competições? “latejando”; Sem instabilidade V2 - Quão instável seu ombro ou cotovelo parece durante a competição? Desloca rotineiramente; Sem instabilidade	4. Quão instável seu ombro ou cotovelo fica durante as competições? Desloca (instável) rotineiramente; Sem instabilidade.
5. How much have arm problems affected your relationship with your coaches, management, and agents? Left team, traded or waived, lost contract or scholarship; Not at all.	V1 - O quanto os problemas do seu braço afetaram seu relacionamento com seus treinadores, empresários e agentes? Saí do time, trocado ou dispensado, perdi contato ou bolsa de estudos; Nem um pouco. V2 - O quanto problemas no braço têm afetado seu relacionamento com seus treinadores, diretores e agentes? Saí do time, fui trocado ou despedido, perdi um contrato ou bolsa de estudos; Nem um pouco	5. O quanto os problemas do seu braço têm afetado seu relacionamento com seus treinadores, gestores e agentes? Saí do time; fui trocado ou despedido; perdi um contrato ou bolsa de estudos; Nem um pouco
The following questions refer to your level of competition in your sport. Please answer with an X along the horizontal line that corresponds to your current level.	V1 - As seguintes perguntas são referentes a sua categoria de competição no seu esporte. Por favor, responda com um X sobre a linha horizontal da forma que você ache que melhor corresponde ao seu nível atual. V2 - As seguintes perguntas são referentes a sua categoria de competição no seu esporte. Por favor, responda com um X sobre a linha horizontal da forma que você ache que melhor corresponde ao seu nível atual.	As seguintes perguntas são referentes a sua categoria de competição no seu esporte. Por favor, responda com um X sobre a linha horizontal da forma que você ache que melhor corresponde ao seu nível atual.
6. How much have you had to change your throwing motion, serve, stroke, etc, due to your arm? Completely changed, don’t perform motion anymore; No change in motion.	V1 - O quanto você teve que mudar seu movimento de arremesso, saque, rebatimento, etc., por causa de seu braço? Mudou completamente, não faço o movimento mais; Sem mudança no movimento. V2 - Quanto você teve que mudar seu movimento de arremesso, saque, rebate, etc, devido ao seu braço? Mudei completamente, não faço mais nenhum desses movimentos; Sem mudanças no movimento	6. O quanto você teve que mudar seu movimento de arremesso, saque, rebatimento, etc., por causa de seu braço? Mudou completamente, não faço mais o movimento mais; Sem mudança no movimento
7. How much has your velocity and/or power suffered due to your arm? Lost all power, became finesse or distance athlete; No change in velocity/power.	V1 - Quanto a sua velocidade e/ou força mudaram por causa de seu braço? Perdi todo o poder, tornei delicado ou atleta de longa distância; Sem mudança na velocidade/força V2 - Quanto sua velocidade e/ou força diminuíram devido ao seu braço? Perdi toda a força, me tornei um atleta de jogos estratégicos ou longa-distância; Sem mudanças na velocidade/força	7. Quanto sua velocidade e/ou força diminuiu devido ao seu braço? Perdi toda a força, alterei minha técnica de arremesso ou deixei de ser um atleta de bolas rápidas e me tornei um arremessador de longa-distância; Sem mudanças na velocidade/força.
8. What limitation do you have in endurance in competition due to your arm? Significant limitation (became relief pitcher, switched to short races for example); No endurance limitation in competition.	V1 - O quão limitada é a sua resistência em competições por causa de seu braço? Limitação significativa (me tornei arremessador reserva, troquei para corrida de curta distância por exemplo); Sem limitação de resistência em competições V2 - Que limitações você tem em relação a resistência em competições devido ao seu braço? Limitações significativas (me tornei substituto, mudei para corridas curtas, por exemplo); Sem limitações de resistência em competições	8. Qual é a limitação da sua resistência em competições por causa de seu braço? Limitação significativa (me tornei arremessador reserva, passei a ter menos tempo de jogo, por exemplo); Sem limitação de resistência em competições
9. How much has your control (of pitches, serves, strokes, etc.) suffered due to your arm? Unpredictable control on all pitches, serves, strokes, etc; No loss of control	V1 - Quanto o seu controle (de arremessos, saques, rebatimento, etc.) sofreu por causa de seu braço? Controle imprevisível em todos os arremessos, saques, rebatimentos, etc.; Sem perda de controle V2 - Quanto seu controle diminui devido ao seu braço? Controle imprevisível em arremessos, saques, rebates, etc; Sem perda de controle	9. Quanto o seu controle (de arremessos, saques, rebatimento, etc.) sofreu por causa de seu braço? Controle imprevisível em arremessos, saques, rebatimentos, etc; Sem perda de controle
10. How much do you feel your arm affects your current level of competition in your sport (ie, is your armholding you back from being at your full potential)? Cannot complete, had to switch sports; Desired level of competition.	V1 - O quanto você sente que o seu braço afeta o seu nível de competição no seu esporte (exemplo, seu braço está lhe impedindo de usar todo o seu potencial)? Não posso competir, tive que trocar de esporte. Nível desejado de competição V2 - Quanto você sente que seu braço afeta o seu nível atual de competição no seu esporte (isto é, seu braço tem te impedido de estar na sua capacidade máxima?) Não consigo competir, tive que mudar de esporte; Estou no meu nível de competição desejado	10. O quanto você sente que o seu braço afeta o seu nível de competição no seu esporte (ou seja, seu braço está lhe impedindo de usar todo o seu potencial)? Não consigo competir, tive que mudar de esporte; Estou no meu nível de competição desejado

V1 = Portuguese version by the first translator; V2 = Portuguese version by the second translator; and V3 = Consensus Portuguese version defined at the end of the initial translation phase.

The consensus version (V3) was back-translated into versions B1 and B2, which are described in **Table 3**. After back-translation, the differences between all versions and the original questionnaire were discussed with the entire committee to culminate in a version with semantic, idiomatic, and cultural equivalence to the original.

**Table 3:** Comparison of back-translations

Original	Backtranslation 1 (B1) and Backtranslation 2 (B2)
Name... Age... Sex... Dominant Hand (R) (L) (Ambidextrous)... Date of Examination... Sport... Position... Years Played	B1 - Name .. Age... Sex...Dominant Hand: (R) (L) (Ambidextrous) Date of test … Sport .. Position .. Years of practice .. B2 - Name… Age… Sex…Dominant hand: (R). (L);(Ambidextrous)... Test date ... Sport... Position ... Years of practice
Please answer the following questions related to your history of injuries to YOUR ARM ONLY:	B1 - Please answer the following questions related to your history of injuries ONLY OF YOUR ARM: B2 - Please answer the following questions regarding your history of injuries TO YOUR ARM ONLY:
1. Is your arm currently injured?	B1 - Is your arm currently injured? B2 - Is your arm currently injured?
2. Are you currently active in your sport?	B1 - Are you currently active in your sport? B2 - Are you currently active in your sport?
3. Have you missed game or practice time in the last year due to an injury to your shoulder or elbow?	B1 - Did you miss any match (game) or training in the last year due to an injury to your shoulder or elbow? B2 - Have you missed any matches (games) or practices in the last year due to an injury to your shoulder or elbow?
4. Have you been diagnosed with an injury to your shoulder or elbow other than a strain or sprain? If yes, what was the diagnosis?	B1 - Have you ever been diagnosed with any shoulder or elbow injury besides strain or sprain? If so, what was the diagnosis? B2 - Have you ever been diagnosed with any shoulder or elbow injuries other than a strain or sprain? If yes, what was the diagnosis?
5. Have you received treatment for an injury to your shoulder or elbow? If yes, what was the treatment? (Check all that apply): Rest... Therapy... Surgery (please describe)	B1 - Have you ever received any treatment for injuries to your shoulder or elbow? If so, what was the treatment? (Check all that apply) Rest; Therapy; Surgery (specify) B2 - Have you ever received any treatment for injuries to your shoulder or elbow? If yes, what was the treatment? (Check all that apply) Rest; Therapy; Surgery (specify)
Please describe your level of competition in your current sport: (Use Professional Major League, Professional Minor League, Intercollegiate, High School as the choices)	B1 - Please, describe your level of competition in your current sport: (Use as options: Professional of Big Leagues, Second-division Professional, Intercollegiate, School) B2 - Please describe your level of competition in your current sport: (Use as options: Major League Pro, Division Two Pro, Intercollegiate, Intramural)
6. What is the highest level of competition you’ve participated at?	B1 - What is the highest level of competition in which you have ever participated? B2 - What is the highest level of competition you have ever participated in?
7. What is your current level of competition?	B1 - At what level are you currently competing? B2 - What level are you currently competing at?
8. If your current level of competition is not the same as your highest, do you feel it is due to an injury to your arm?	B1 - If your current level of competition is not the same as the highest at which you have ever competed, do you feel that it is due to the injury to your arm? B2 - If your current level of competition is not the same as the highest you have ever competed in, do you feel it is due to your arm injury?
Please check the ONE category only that best describes your current status: Playing without any arm trouble... Playing, but with arm trouble... Not playing due to arm trouble	B1 - Please check only ONE option that best describes your current situation: Playing with no problem in the arm; Playing, but with a problem in the arm; Not playing because of a problem in the arm; B2 - Please check only ONE option that best describes your current situation: Playing without any arm problem; Playing but with an arm problem; Not playing because of an arm problem;
Instructions to athletes: The following questions concern your physical functioning during game and practice conditions. Unless otherwise specified, all questions relate to your shoulder or elbow. Please answer with an X along the horizontal line that corresponds to your current level.	B1 - Instructions for athletes: The following questions refer to your physical functioning during games and training. All questions are related to your shoulder or elbow unless it is otherwise specified. Please, answer with an X on the space of the horizontal line that best corresponds to your current level. B2 - Instructions for athletes: The following questions refer to your physical functioning during games and practices. All questions relate to your shoulder or elbow unless otherwise specified. Please answer with an X over the space on the horizontal line that best corresponds to your current level.
1. How difficult is it for you to get loose or warm prior to competition or practice? Never feel loose during games or practice; Normal warm-up time	B1 - How difficult is it for you to warm up or get loose (relax) before a competition or training? Never feel loose (relaxed) during games or training Normal training time B2 - How difficult is it for you to warm up or loosen up (relax) before a competition or practice? Never feel loose (relaxed) during games or practice Normal Warm-up time
2. How much pain do you experience in your shoulder or elbow? Pain at rest; No pain with competition	B1 - How much pain do you feel in your shoulder or elbow? Pain at rest No pain in competition B2 - How much pain do you feel in your shoulder or elbow? Pain when resting No pain during the competition.
3. How much weakness and/or fatigue (ie, loss of strength) do you experience in your shoulder or elbow? Weakness or fatigue preventing any competition; No weakness, normal competition fatigue	B1 - How much weakness and/or fatigue (that is, loss of strength) do you feel in your shoulder or elbow? Weakness or fatigue impedes me from competing No weakness, I feel a normal fatigue of competition B2 - How much weakness and/or fatigue (loss of strength) do you feel in your shoulder or elbow? Weakness or fatigue that prevents me from competing No weakness, I feel normal competition fatigue.
4. How unstable does your shoulder or elbow feel during competition? “Popping out” routinely No instability	B1 - How unstable does your shoulder or elbow get during competitions? Dislocates (unstable) routinely; No instability B2 - How unstable is your shoulder or elbow during competitions? It is displaced (unstable); No instability
5. How much have arm problems affected your relationship with your coaches, management, and agents? Left team, traded or waived, lost contract or scholarship; Not at all.	B1 - How much have the problems in your arm affected your relationship with trainers, managers, and agents? I left the team, I was traded or let go; I lost a contract or scholarship. Not at all B2 - How much have your arm problems affected your relationships with your coaches, managers, and agents? I left the team; I was traded or fired; I lost a contract or scholarship. Not at all.
The following questions refer to your level of competition in your sport. Please answer with an X along the horizontal line that corresponds to your current level.	B1 - The following questions are referent to your competition category in your sport. Please, answer with an X on the horizontal line in the way that you think best corresponds to your current level. B2 - The following questions refer to your competition category in your sport. Please answer with an X over the horizontal line, the way you think best corresponds to your current level.
6. How much have you had to change your throwing motion, serve, stroke, etc, due to your arm? Completely changed, don’t perform motion anymore; No change in motion.	B1 - How much did you have to change your throwing (pitching), serving, batting, etc. because of your arm? Changed completely, I no longer do the movement. No change in the movement. B2 - How much did you have to change your throwing, serving, batting, etc movements because of your arm? Completely changed, I don’t make the move anymore No change in the movement.
7. How much has your velocity and/or power suffered due to your arm? Lost all power, became finesse or distance athlete; No change in velocity/power.	B1 - How much has your speed and/or strength diminished due to your arm? I lost all strength, I changed my throwing technique or I quit being a fastball athlete and became a long-distance thrower No changes in speed/strength B2 - How much has your speed and/or strength decreased because of your arm? I lost all power, I changed my throwing technique or went from being a fast-ball athlete to a long-distance thrower. No changes in speed/strength.
8. What limitation do you have in endurance in competition due to your arm? Significant limitation (became relief pitcher, switched to short races for example); No endurance limitation in competition.	B1 - What is the limitation of your endurance during competitions because of your arm? Significant limitation (I became a reserve pitcher, I switched to short-distance running, for example). No limitation of endurance in competitions B2 - What is the limitation of your endurance in competitions because of your arm? Significant limitation (I became a backup pitcher, and switched to short races, for example). No endurance limitation in competitions.
9. How much has your control (of pitches, serves, strokes, etc.) suffered due to your arm? Unpredictable control on all pitches, serves, strokes, etc; No loss of control	B1 - How much has your control (of throws (pitches), serves, batting, etc.) suffered because of your arm? Unpredictable control of throws, serves, bats, etc. No loss of control B2 - How much has your control (of throwing, serving, hitting, etc.) suffered because of your arm? Unpredictable control on throws, serves batting, etc. No loss of control.
10. How much do you feel your arm affects your current level of competition in your sport (ie, is your arm holding you back from being at your full potential)? Cannot complete, had to switch sports; Desired level of competition.	B1 - How much do you feel that your arm affects your level of competition in your sport (that is, is your arm impeding you from using your full potential)? I am not able to compete, I had to change sports I am at my desired level of competition B2 - How much do you feel that your arm affects your level of competition in your sport (is your arm preventing you from using your full potential)? I’m not able to compete, I had to change sports. I am at my desired competition level.

B1 = version from the first back translator; B2 = English version from the second back translator.

During the pre-test, all items achieved comprehensibility above 90%, and there were no suggestions for removing or adding questions to the questionnaire. However, during the observation of the responses, it was noticed that a few athletes had difficulty understanding the horizontal line. Therefore, in the discussion with the expert committee, the 10 cm horizontal line was replaced by an 11-point numerical rating scale to optimize the understanding of these items. Additionally, a few athletes suggested changing question 1 from “Tempo normal de aquecimento” to “Se sente solto (relaxado(a)) durante tempo normal de aquecimento”, and question 7 from “Perdi toda a força, alterei minha técnica de arremesso ou deixei de ser um atleta de bolas rápidas e me tornei um arremessador de longa-distância” to “Perdi toda a minha força e velocidade. Tive que alterar minha técnica”. The final version of the questionnaire is presented in **Supplementary Material 1** https://osf.io/9puxg/?view_only=05246b5e7b3e48aba5aedf3fdc37e114.

## DISCUSSION

The translation and cultural adaptation of the KJOC questionnaire for use in Portuguese was conducted, and a Portuguese version for use in Brazil was developed. This process followed the methods recommended by Beaton et al.^32^, previously used in the translation and adaptation of various questionnaires in various countries, such as the ABILHAND-Kids, the revised Foot Function Index (FFI-R), the Bournemouth questionnaire,^38,39,40^ and the KJOC questionnaire.^17,25^

Throughout the process, the primary translations and subsequent back-translations into English resulted in minor linguistic inconsistencies that were resolved during the synthesis group and committee meetings. A few terms were translated non-literally to achieve cultural equivalence with the study population. For example, the phrase “switched to short races” in question eight was originally translated to “corrida de curtas distâncias” (short-distance race). During the discussion, it was identified that the term did not make sense in Portuguese; therefore, it was modified to “passei a ter menos tempo de jogo” (started to have less playing time) to make it understandable and maintain its original meaning. Moreover, there were changes regarding the adaptation of the American Sports Classification system to the Brazilian system in question 6, which resulted in the translation of ‘Professional Minor League’ to “Profissional da Segunda Divisão” (Second Division Professional), for example. Additionally, a numerical rating scale was added, as previously reported, to facilitate athletes’ understanding of how to complete the questionnaire.^18^ Question 7 was revised to “Perdi toda a minha força e velocidade,” as reported in another translation and cultural adaptation.^17^

After the modifications, the questionnaire was subjected to a pre-test application, a practice commonly adopted in similar studies, such as those conducted by Schulz et al.^25^ and Sukanen et al.,^14^ who applied a preliminary version of the questionnaire to 10 athletes. In the Brazilian context, this approach is already widely used and plays a fundamental role in the cultural adaptation process, as it allows for the assessment of how the target population interprets the questionnaire items.^40^ The pre-test version was employed to ensure that the questionnaire was understood by more than 90% of the participants. Some suggestions from the athletes improved its readability, thereby enabling the questionnaire to be culturally adapted appropriately for the Brazilian population.^30^

Similar to other translations and cultural adaptations of the KJOC questionnaire,^14,17^ no difficulties were encountered in applying the Brazilian version. Athletes identified the questionnaire as easy to understand and respond to, with no need for further modifications. Previous studies have reported that athletes respond to the KJOC questionnaire in approximately 10 minutes.^16,18^ Unfortunately, we did not measure the duration that athletes took to respond to the Brazilian version of the KJOC questionnaire. Despite guidelines recommending that reliability and validation analysis of a measurement instrument necessarily occur after translation and cultural adaptation,^32^ studies have been conducted and published only with the translation and cultural adaptation process.^21,40,41,42^ This is done to avoid multiple translations and versions of the same instrument, thus preventing unnecessary work.^38^ Test-retest reliability of other versions of the KJOC questionnaire has been investigated and demonstrated excellent results, showing good internal consistency and the ability to detect clinically significant improvements in patients.^16,17,25^ Thus, the psychometric properties of the Brazilian Portuguese version are being evaluated, and it is expected that the results will contribute to its application in Brazil.

The clinical and research relevance of this study lies in its contribution to providing another assessment tool for shoulder and elbow function in overhead athletes. This questionnaire offers valuable insights for both therapists and researchers, aiding in the collection of information necessary for rehabilitation practices by identifying specific functional impairments and tracking rehabilitation progress over time.

## CONCLUSION

The translation and cultural adaptation of the KJOC shoulder and elbow questionnaire into Portuguese were completed, and a Brazilian version was obtained. Future studies are needed to verify the measurement properties of the Brazilian version of the questionnaire.
